# Pancreas-Preserving Approach to “Paraduodenal Pancreatitis” Treatment: Why, When, and How? Experience of Treatment of 62 Patients with Duodenal Dystrophy

**DOI:** 10.1155/2014/185265

**Published:** 2014-06-05

**Authors:** V. I. Egorov, A. N. Vankovich, R. V. Petrov, N. S. Starostina, A. Ts. Butkevich, A. V. Sazhin, E. A. Stepanova

**Affiliations:** ^1^Department of Surgical Oncology, Moscow City Hospital No. 5, Sechenov First Moscow State Medical University, Stromynka Street 7, Moscow 107076, Russia; ^2^Hepatopancreatobiliary Department, Vishnevsky Institute of Surgery, Bolshaya Serpukhovskaya Street 27, Moscow 117997, Russia; ^3^General Surgery Department, Central Hospital of FSS RF, Petrovskoye Schosse 48, Golitsino, Moscow 143040, Russia; ^4^General Surgery Department, Moscow City Hospital No. 4, N. Pirogov Russian National Research Medical University, Ostrovityanova Street 1, Moscow 117513, Russia; ^5^Department of Pathology, Moscow City Hospital No. 12, Bakinskaya Street 26, Moscow 115516, Russia

## Abstract

*Background*. The term “paraduodenal pancreatitis” (PP) was proposed as a synonym for duodenal dystrophy (DD) and groove pancreatitis, but it is still unclear what organ PP originates from and how to treat it properly. *Objective*. To assess the results of different types of treatment for PP. *Method*. Prospective analysis of 62 cases of PP (2004–2013) with histopathology of 40 specimens was performed; clinical presentation was assessed and the results of treatment were recorded.  *Results*. Preoperative diagnosis was correct in all the cases except one (1.9%). Patients presented with abdominal pain (100%), weight loss (76%), vomiting (30%), and jaundice (18%). CT, MRI, and endoUS were the most useful diagnostic modalities. Ten patients were treated conservatively, 24 underwent pancreaticoduodenectomies (PD), pancreatico- and cystoenterostomies (8), Nakao procedures (5), duodenum-preserving pancreatic head resections (5), and 10 pancreas-preserving duodenal resections (PPDR) without mortality. Full pain control was achieved after PPRDs in 83%, after PDs in 85%, and after PPPH resections and draining procedures in 18% of cases. Diabetes mellitus developed thrice after PD. *Conclusions*. PD is the main surgical option for PP treatment at present; early diagnosis makes PPDR the treatment of choice for PP; efficacy of PPDR for DD treatment provides proof that so-called PP is an entity of duodenal, but not “paraduodenal,” origin.

## 1. Introduction

Cystic dystrophy of the duodenal wall, or duodenal dystrophy (DD), is a relatively rare disease which is essentially a chronic inflammation of ectopic (aberrant, heterotopic) pancreatic tissue in the duodenal wall. This entity was first described in 1970 by French authors Potet and Duclert [1], and “duodenal dystrophy” as a term for this condition was also proposed by these authors.

Despite genetic predisposition, heterotopic pancreatic tissue in any abnormal location is usually diagnosed in adults presenting with complications. Chronic pancreatitis developing in the intraduodenal ectopic pancreas is characterized by fibrotic thickening and infiltration of the duodenal wall (typically, its vertical branch) with cyst formation in its muscle and/or submucosal layers. Initially, only ectopic pancreatic tissue may be involved, but progressing ectopic pancreatitis may result in the compression of the main pancreatic or accessory pancreatic duct and subsequent obstructive pancreatitis in the orthotopic (main) pancreas [1–7]. There is still uncertainty with the terminology for this condition, and it is rooted in an uncertain localization of the primary lesion. For example, “groove pancreatitis” and DD are considered synonyms by some authors [5], whereas others believe that DD is one of the causes of “groove pancreatitis” (groove pancreatitis is a form of focal chronic pancreatitis in the pancreatic tissue between the duodenal wall and the intrapancreatic portion of the common bile duct (CBD) [8]). DD is classified into cystic or solid types according to predominating component (fibrotic thickening or cyst formation) [6, 7, 9–12]. Against the background of chronic inflammation in the orthotopic pancreas, it is difficult to confirm DD of the solid type, and, hence, cystic variant is found much more often, so the diagnosis of DD generally implies its cystic form. Cysts of the ectopic pancreas can be either postnecrotic or represented by a cystically dilated bile duct with preserved or desquamated epithelium [6–8]. More often (but not necessarily), the disease occurs against the backdrop of regular alcohol consumption. In 15 retrospective reports, 79.68% (251 out of 305) patients with duodenal dystrophy were alcohol abusers [13].

Duodenal dystrophy is typically manifested by recurrent episodes of acute pancreatitis, recurrent or chronic abdominal pain in the epigastrium, left, or right upper quadrant, weight loss, and nausea and vomiting caused by the duodenal stenosis. As the pathological process in the orthotopic pancreas progresses, the clinical picture is becoming more similar to that of chronic pancreatitis [4, 5]. Pancreatic ectopy within the gastric wall (25–60%) and duodenum (25–35%) is the most common gastrointestinal heterotopia [13, 14]. Nevertheless, DD is found relatively infrequently [9–12]. The instrumental semiotics of DD is well studied: the diagnosis is based on CT or MRI imaging and endosonography [10–15]. The conservative treatment is based on the use of regular or long-lasting somatostatin analogues [16–18] and can be complemented with endoscopic manipulations [19, 20]. If the above approaches fail, surgical procedures are considered, and pancreaticoduodenectomy (PD) remains the method of choice [21–33]. When DD is complicated by obstructive jaundice, duodenal stenosis, and chronic orthotopic pancreatitis with its typical complications, as well as in cases of suspected tumor [34, 35], the conservative treatment [36, 37] is either* a priori* ineffective or not indicated.

The aim of this prospective study was to analyze the clinical and demographic characteristics of patients and methods of DD diagnosis and treatment.

In 2004, Adsay and Zamboni [5] suggested a term “paraduodenal pancreatitis” for the duodenal dystrophy, and it has become widespread. However, we refrained from such renaming based on our own data definitely indicating the localization of the pathology in the duodenum but not in the “paraduodenal” area (see Sections 3 and 4).

## 2. Patients and Methods

Sixty-two patients with DD were evaluated, treated, and followed up by the authors at the Moscow hospitals mentioned in authors affiliations during 2004–2013. All patients were symptomatic and demonstrated characteristic signs of DD on CT, MRI, and endosonography. By January 2013, 52 patients had undergone surgical treatment. The histological diagnosis of the cystic type of DD was based on the detection of pancreatic tissue isolated from the orthotopic gland and/or cystic masses in the duodenal wall with elements of transformed pancreatic tissue. When the duodenum was not available for histological examination, the diagnosis was based on the CT, MRI, or endosonographic findings on the ground of the pathognomonic signs: significant (>10 mm) thickening of the duodenal wall, cystic masses of variable size within the duodenal wall [10–13, 32], demarcation of pathological changes within the duodenal wall from the orthotopic pancreas, and medial displacement of the gastroduodenal artery from the pathological mass.

Clinical characteristics, pathological findings, data of various imaging techniques, and intraoperative, postoperative, and follow-up data were recorded for all patients. Clinical data included patient's age, gender, alcohol consumption, date of diagnosis, symptoms (weight loss or weight gain, vomiting, abdominal pain, jaundice, and steatorrhea), and enzyme therapy. Laboratory tests included C-reactive protein, fibrinogen, ESR, creatinine, electrolytes, bilirubin, AP, GGTP, ALT, and AST. Imaging data were evaluated by an attending surgeon jointly with a radiologist and gastroenterologist.

### 2.1. Procedures

The description of standard* pylorus preserving pancreaticodudenectomy* can be found elsewhere. As draining procedures we used pancreaticojejuno or cystopancreaticojejunostomy. As duodenum-preserving operations, the subtotal pancreatic head resection under the names of Bern [38] or Frey procedure [39] was used, which means the same amount of resected pancreatic tissue after modifications described by Frey and Mayer in 2003 [39].

*Total pancreatic head resection with segmental duodenectomy* including minor and major papilla (*Nakao procedure*) was performed by conserving the right gastric artery and the anterior inferior pancreaticoduodenal artery. Five to seven cm of the first portion, the third portion, and the anal side of the second portion of the duodenum is preserved with good arterial circulation. Reconstruction of the alimentary tract is then performed with pancreatogastrostomy, end to end duodenoduodenostomy, and end to side choledochoduodenostomy [40].

**Figure 1:**
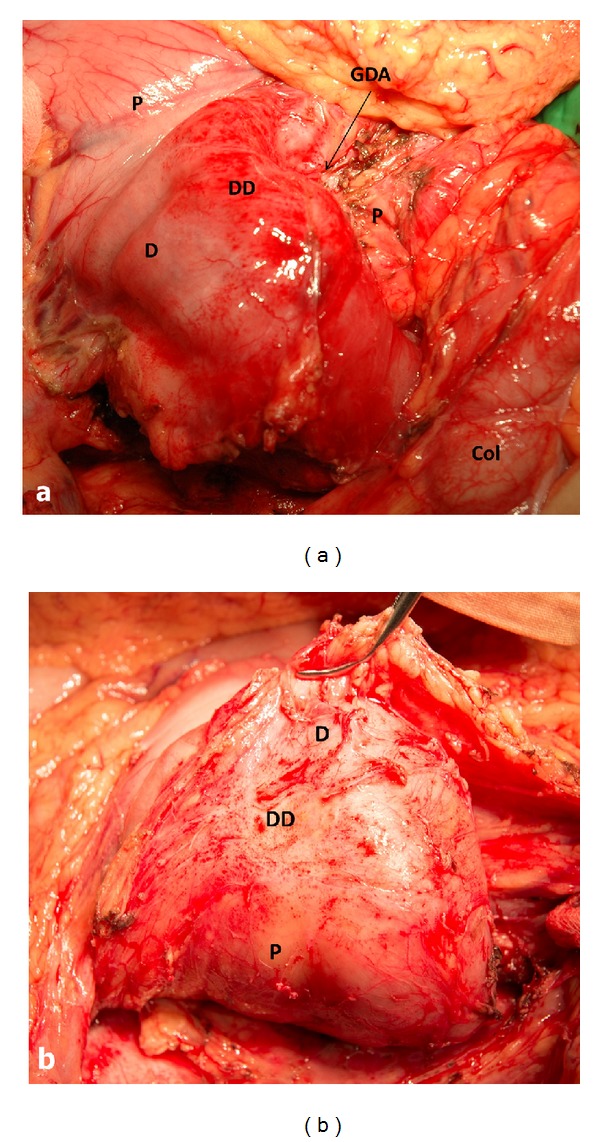
Isolated form of the duodenal dystrophy (DD). Male, 57 y.o. Itraoperative photo. (a) Front view. Kocher's maneuver is completed. Deformation, hyperemia, and thickening of the medial duodenal wall with infiltrated fibrotic tissues around the duodenum (D). The gastroduodenal artery (GDA) is shifted forward and medially, lying in the groove between the unchanged pancreatic head (P) and affected duodenal wall. (b) Back view. Extensive Kocher's maneuver is completed. The duodenum and pancreatic head (P) look like inseparable monolith due to prominent fibrosis around the second portion of the duodenum (D). Col—transverse colon, Py—pylorus.

*Pancreas*-*preserving operations for duodenal dystrophy* begin through the midline incision with exploration and an extensive Kocher's maneuver well to the left. After detection of the inflammatory mass in the second part of the duodenum (Figures 1(a) and 1(b)), cholecystectomy is performed and the papilla is stented through the cystic duct stump with a Dogliotti probe as a landmark.

**Figure 2:**
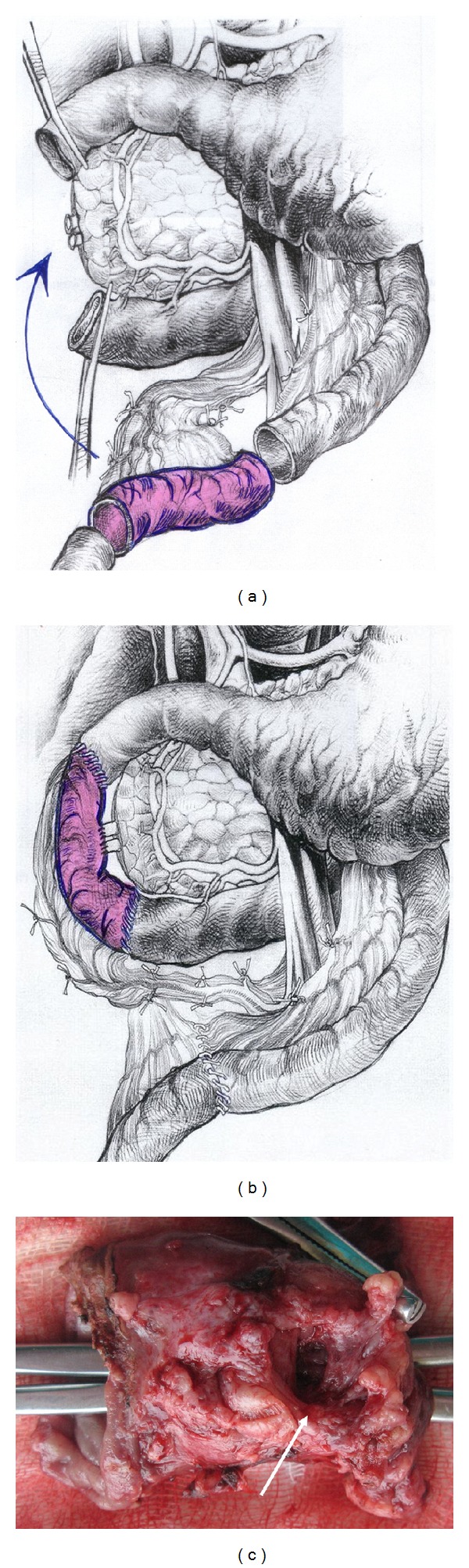
Duodenal dystrophy with moderate chronic orthotopic pancreatitis. Patient, 32 y.o. Scheme of the pancreas-preserving resection of the second portion of the duodenum. (a) The second part of the duodenum, including the main papilla, is removed and the segment of the proximal jejunum supplied by the artery and vein is cut out and prepared for transposition between the 1st and 3rd portions of the duodenum; (b) the shifted segment is interposed between the 1st and the 3rd parts of the duodenum. Jejuno-jejuno- and duodeno-jejuno-anastomoses are performed. The bile and the pancreatic ducts were implanted in the neoduodenum 4 cm below the proximal duodeno-jejuno-anastomosis; (c) the resected specimen of the second part of the duodenum. A large scarry-sided cyst in the medial duodenal wall is shown (arrow). Forceps were introduced into the duodenum to show the absence of communication between the cystic and duodenal lumen.

**Figure 3:**
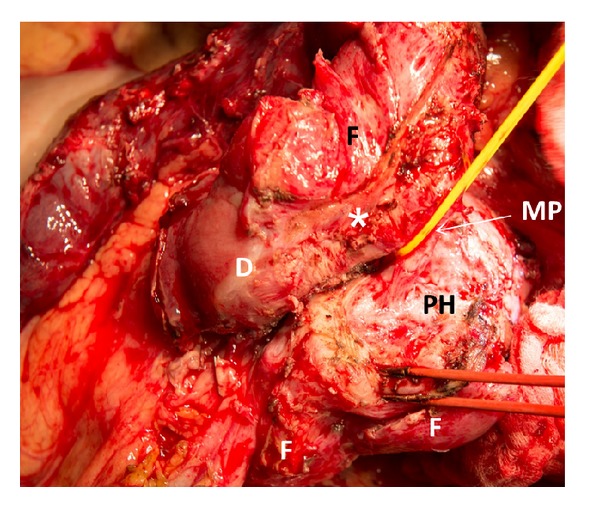
Isolated form of the duodenal dystrophy (DD). Male, 57 y.o. Intraoperative photo. Front view. Kocher's maneuver is completed. The first, third, and fourth portions of the duodenum (D) are detached from the pancreas head (PH) without its injury. The duodenal cyst located around the major papilla (MP) was opened and the papilla was taken by yellow tape. The fibrosis (F) was marked and medial cystic wall was left on the pancreas head (PH) in order not to damage it. There is no pancreatic tissue attached to the duodenal wall (∗).

When the length of affected zone is short its* resection with duodenoduodenostomy* is possible but the probability of tension is a limitation of such a method. If the length of inflammatory area is not spread beyond the second duodenal portion the surgeon can choose its* resection with intestinal interposition for reconstruction* (Figures 2(a), 2(b), and 2(c)). In spite of moderately changed or unchanged pancreas, very often at surgery the duodenum and pancreatic head look inseparable due to prominent fibrosis around the duodenum (Figures 1 and 3) and this is the main difference between the corresponding procedures for familial adenomatous polyposis. The duodenum is transected 2-3 cm below the pylorus and 3 cm below the main papilla. The second portion of the duodenum is detached from the pancreas by division of the short vessels by ultrasound scissors up to major papilla. Usually, during this detachment at the level of the major papilla, intramural duodenal cyst(s) is(are) opened and its(their) form(s) and location can be different: some of them are placed along one side of papilla vateri and some are surrounding it (Figure 3). Following the transection of the common bile and the main pancreatic ducts and detachment of the duodenum from the pancreatic head, the whole of its second part, including the main papilla, is removed (Figure 2(a)). In the case of direct duodeno-duodenal anastomosis it forms by end-to-end-technique. In case of jejunal pouch method, a 10 cm segment of the proximal jejunum, supplied by the artery and vein, 50 centimeters below the Treitz ligament, is cut out and passed through the mesocolon (Figure 2(a)). The shifted segment is interposed between the first and the third parts of the duodenum and jejuno-jejuno- and distal duodeno-jejuno-anastomoses are performed (Figure 2(b)). When the inflammation and scarring are not so pronounced, it is possible to remove all the cystic walls without injury of the pancreas (Figure 2(c)); if the fibrosis is marked it is better to leave medial cystic wall on the pancreas in order not to damage it (Figure 3). The latter is safe with regard to possible relapse because the cysts have no epithelium due to long-term inflammation. Frozen section of the removed duodenum is mandatory to exclude cancer.

**Figure 4:**
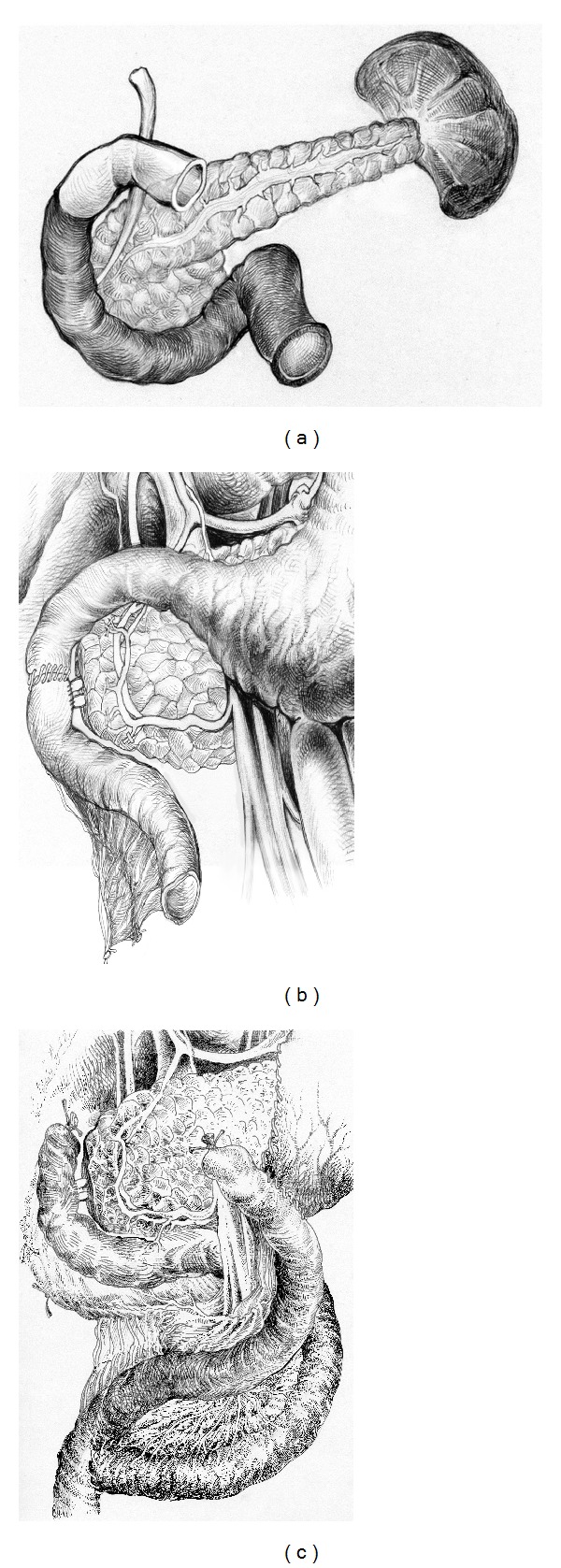
Duodenal dystrophy with moderate chronic orthotopic pancreatitis. Patient, 43 y.o. Scheme of the pancreas-preserving subtotal duodenectomy: (a) The parts of the duodenum to be removed are shown in black; (b) completion of the procedure by replantation of pancreatic and common bile ducts into the jejunum 1–1.5 cm below the duodenojejunoanastomosis; (c) patient 49 y.o. Variant of the completion of the pancreas-preserving subtotal duodenectomy with distal gastrectomy by Roux-en-Y reconstruction in case of stomach involvement or peptic ulcer.

**Figure 5:**
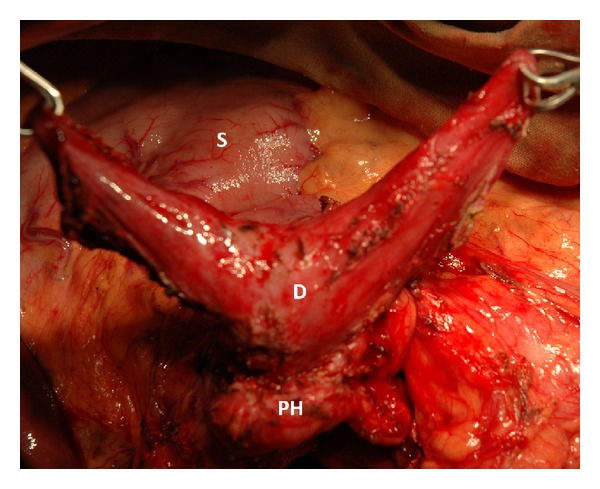
Isolated form of the duodenal dystrophy. Male, 57 y.o. Intraoperative photo. Side view. Kocher's maneuver is completed. Duodenum (D) was transected at the level of ligament of Trietz and 3 cm below the pylorus. The first, third, and fourth portions of the duodenum are detached from the pancreas by division of the short vessels by ultrasound scissors up to the level of the major papilla. S—stomach.

If inflammation and fibrosis of the duodenal wall expand beyond the second duodenal portion, the* subtotal duodenectomy* is preferable (Figures 4(a)–4(c)). Duodenum is transected 2-3 cm below the pylorus. The ligament of Trietz is incised, and the proximal jejunum is transected with the gastrointestinal stapler or by cautery and detached from its short mesentery. The freed jejunum is transferred to the right, behind the superior mesenteric vessels, and the third and fourth portions of the duodenum are detached from the pancreas by division of the short vessels between suture ligatures or by ultrasound scissors up to the level of the major papilla (Figure 5).

The proximal jejunum, mobilized by division of one or two jejunal branches but preserving the arcades, is passed either behind or in front of the superior mesenteric vessels for an end-to-end (Figure 4(b)) or Roux-en-Y anastomosis with the duodenum (Figure 4(c)). If cyst spreads up to stomach wall or there is a peptic duodenal or gastric ulcer the procedure can be added by distal gastrectomy with subsequent Roux-en-Y gastroenterostomy.

**Figure 6:**
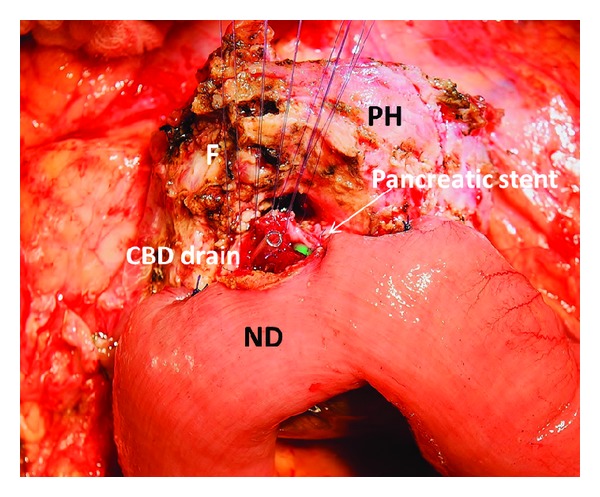
Isolated form of the duodenal dystrophy (DD). Male, 57 y.o. Itraoperative photo. Front view. The subtotal duodenectomy is performed: the first, third and fourth portions of the duodenum are detached from the pancreas head (PH) and removed. Jejunum transferred from below is becoming the neoduodenum (ND). The posterior wall of the choledocho-pancreatico-jejunostomy is sutured by duct to mucosa technique. The tip of the common bile drain duct and stent in the narrow pancreatic duct are visible. Marked fibrotic massif (F) surrounds the pancreatic head (PH) and will be used to cover the front wall of the anastomosis.

The papilla has no landmarks except the Dogliotti probe which helps to identify it around “fibrotic fields.” The common bile duct and the bounded to its inferior aspect pancreatic duct are transected. If narrow the pancreatic duct can be intubated with a 1.3 mm stent the procedures are completed by reconstruction of bile and pancreatic ducts, which are sutured together and implanted in the duodenum 3 cm below direct duodenal anastomosis or in the neoduodenum 4 cm below the duodenojejunostomys (Figures 2 and 4). All the bowel anastomoses are made using a single layer continuous 4/0 absorbable suture. The choledocho-pancreaticojejunostomy is carried out with a single layer of interrupted 5/0 absorbable sutures by duct-to-mucosa technique (Figure 6). We used drainage of the common bile duct through the cystic duct stump and drainage of the upper right abdominal quadrant.

The* conservative treatment* included abstaining from alcohol, administration of analgesics, proton pump inhibitors, somatostatin analogues, nutritive support (parenteral and/or tube feeding), additional endoscopic procedures, or ultrasound-guided punctures and biopsies. Surgery was performed after failure of conservative therapy or occurrence of complications.

The results of treatment were followed up for a period of 12 to 58 months (median 19 months). Body weight and body mass index were measured at baseline, at presentation, and 12 months after surgery, that is, when most notable body weight changes are observed. At later terms, body weight variations were insignificant in all patients. Preoperative weight loss was recorded from patients' medical histories. The amount of pure alcohol consumed was calculated based on patient's information and might have been underestimated. All patients received pancreatin microgranules (Creon) at doses eliminating diarrhea.

The prevalence of pancreatic ectopy within the duodenal wall was estimated from macro- and microscopic findings of 100 sequential autopsies after nonabdominal deaths at the Moscow City Clinical Hospital No. 12.

### 2.2. Statistical Analysis

Statistica software (data analysis software system, version 8.0 StatSoft, Inc. 2001; MedCalc version 11.6.0.0) was used for the statistical analysis. Descriptive statistics were applied with absolute and relative frequencies. Fisher exact test was used for the comparison of the efficacy of the treatment methods. Two-sided *P* values were always computed, and an effect at *P* < 0.05 was considered statistically significant. The distributions of age at operation, alcohol abuse, weight, and BMI are described as medians with interquartile ranges. The numbers of the complications in the groups are expressed as integers without percentage in case of small sample size. Data values are presented on a continuous scale, but distributions different from normal (e.g., patient age) were compared using the Kruskal-Wallis test. One-way analysis of variance was used to test the difference between the means for several groups. Prior to the ANOVA test, Levene's test for equality of variances was performed. The results are presented in an ANOVA graph and associated *P* values if the means for at least two of the groups differ significantly.

## 3. Results

Ectopic pancreatic tissue in the medial duodenal wall was found in three of 100 pancreaticoduodenal specimens from subjects who died of nonabdominal diseases and in none of these cases where the ectopic pancreas was associated with the orthotopic gland. Minor duodenal papilla was not found in two cases, and in one case it was located in the vicinity of the ectopic gland. No alterations were found in the heterotopic tissue, in the orthotopic gland, and in the surrounding tissues.

Duodenal dystrophy was diagnosed in 41 (12.7%) of 323 patients undergoing surgery for chronic pancreatitis in HPB department of Vishnevsky Institute of Surgery in 2005–2011, one of the study sites.

Our series included 59 males and 3 females aged 28 to 73 years (mean age 45.3 years); 57 patients (92%) regularly consumed alcohol (Table 1). Disease duration prior to the diagnosis was 1 to 168 months. Preoperatively, pancreatitis was diagnosed in all patients, with the exception of two women with no history of alcohol consumption and with a suspected cystic tumor of the pancreatic head. All patients were symptomatic at presentation. Weight loss was found in 57 (90%) patients (on average 15.1 kg, range 4 to 30 kg), vomiting was reported by 12 (20%), and jaundice was present in 8 (11%) patients. Cholestasis without jaundice was found in 10 (16%) patients. Nine (14.5%) patients had acute pancreatitis within 3 or less months before diagnosis. Forty (64%) patients had one symptom of the disease, and 21 (33.5%) patients had two or more symptoms (Table 2).

**Figure 7:**
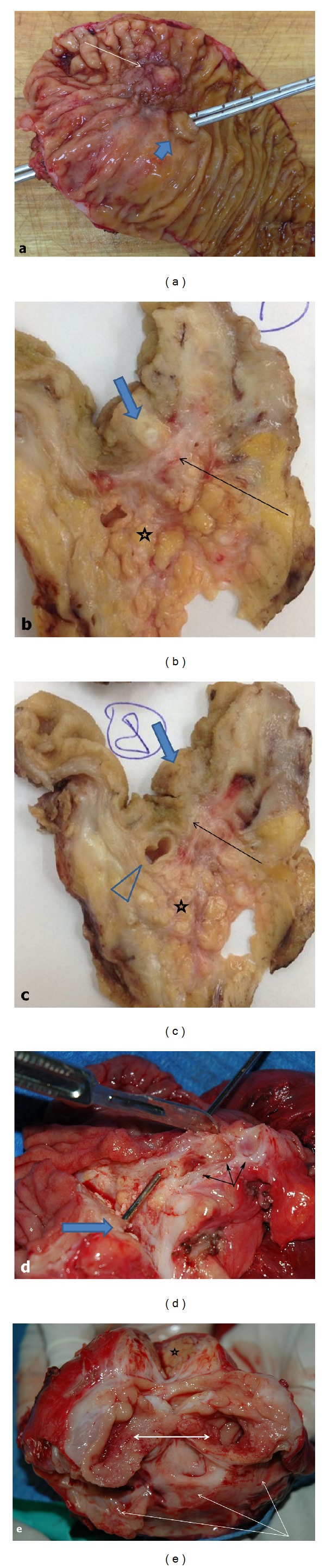
Removed pancreaticoduodenal specimen. (a) Patient 53 y.o. Duodenal dystrophy with chronic orthotopic pancreatitis. Ectopic pancreas within the medial wall of the duodenum (arrow) 1 cm from the main duodenal papilla (wide arrow) with a probe passed through the common bile duct and pancreatic duct; (b) and (c) macrophotograph. Section through the ectopic pancreas. The duodenal wall (arrow) separates the ectopic gland (wide arrow) and the head of the orthotopic pancreas (asterisk) with severe chronic inflammation. The ampulla of Vater (arrowhead) is 0.5 cm from the heterotopic gland; (d) patient 43 y.o. Duodenal dystrophy with moderate chronic pancreatitis in the main pancreas. The probe is passed through the ampulla of Vater. Septated cysts 0.5–1.5 cm in diameter (triple arrow) in the duodenal wall are isolated from the head of pancreas; (e) patient 34 y.o. Duodenal dystrophy with moderate chronic pancreatitis in the main pancreas (asterisk). The second portion of the duodenum is transversely dissected (two-headed arrow). The cyst up to 5 cm in diameter is spread along the whole duodenal wall (triple arrow).

**Figure 8:**
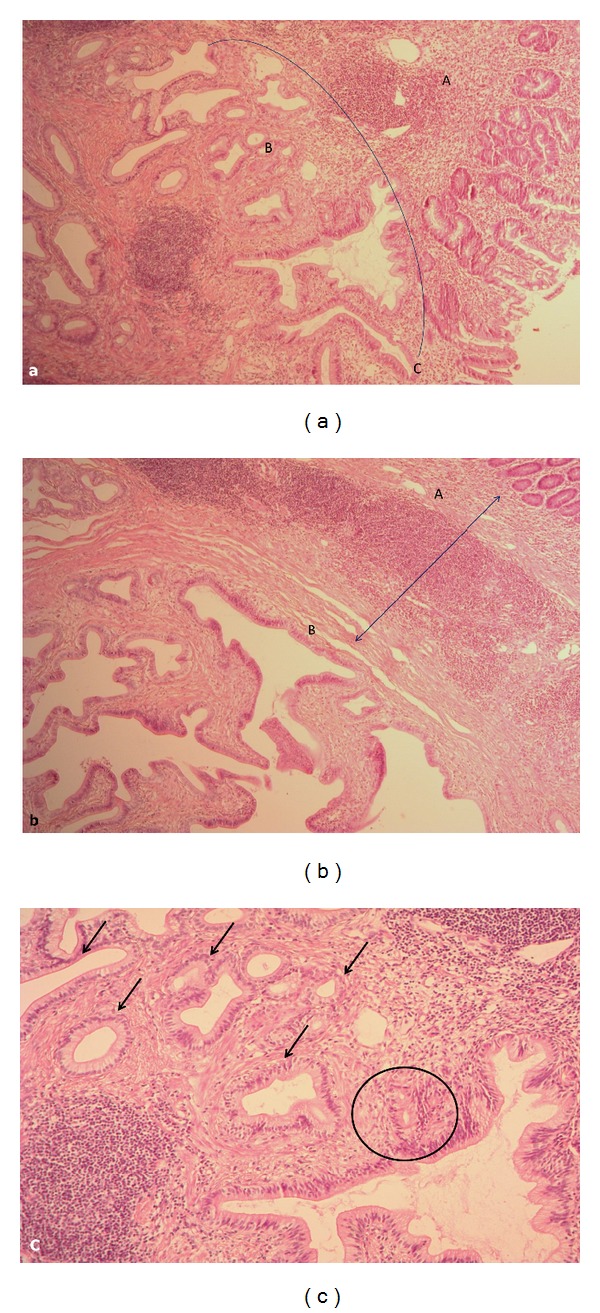
Microphotograph. Patient 53 y.o. Duodenal dystrophy with chronic orthotopic pancreatitis. (a) Ectopic pancreatic tissue (В) in the duodenal wall (A) is presented by acinar-ductal transformation and reaches duodenal mucosa lamina propria (C). Hematoxylin-eosin, ×50; (b) fibrosis and inflammatory infiltration in the ectopic pancreatic tissue in the duodenal wall (B) and at the periphery. Hematoxylin-eosin, ×50; (c) prominent atrophy of the acinar tissue with duct transformation (black arrows) and adenomatous hyperplasia of the epithelium (circle). Hematoxylin-eosin, ×100.

**Figure 9:**
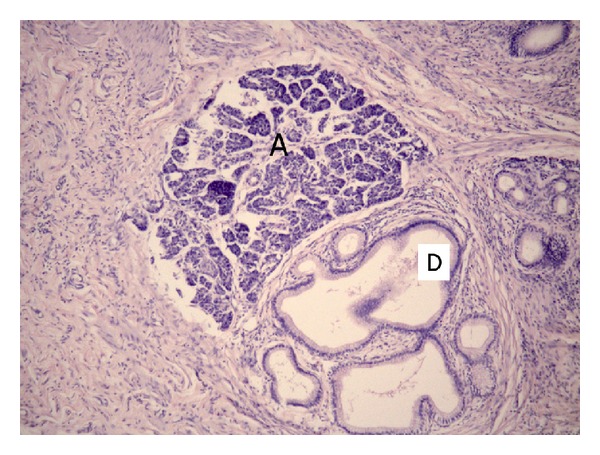
Microphotograph. Patient 47 y.o. Heterotopia of the pancreas tissue (acinuses—A and ducts—D) in the duodenal wall. Hematoxylin-eosin, ×100.

**Figure 10:**
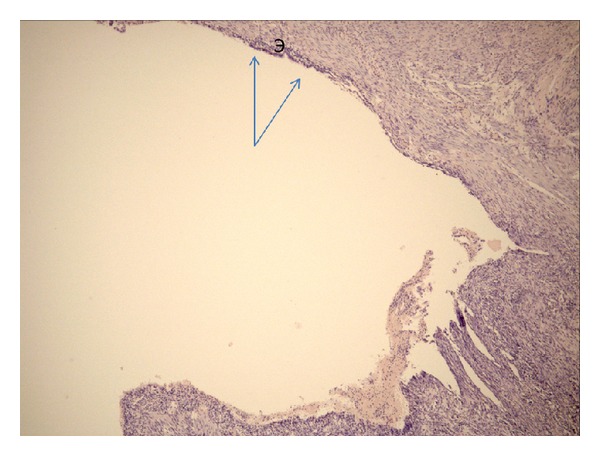
Microphotograph. Patient 61 y.o. Cyst in the duodenal wall formed by a dilated duct of the ectopic gland with islands of preserved epithelium (E and arrows). Hematoxylin-eosin, ×50.

In 39 cases, the diagnosis was confirmed by histopathology of the removed pancreaticoduodenal or duodenal specimens. Typically, the ectopic tissue was found in the muscle layer and, when the size of the mass was large enough, also in the submucosal layer of the duodenum in close proximity to and often involving the major duodenal papilla (60 cases) (Figures 7, 8, and 9). The minor duodenal papilla was not detectable in the majority of cases, but clearly discernible outside the pathological lesion in 5 cases. Cysts could be lined with secretory pancreatic epithelium or composed of fibrotic tissue with polymorphic cell infiltration (Figures 9 and 10). When duodenum-preserving pancreatic head resection or draining procedures were performed, pathohistological examination of pancreatic tissue only was possible. A severe chronic “orthotopic” pancreatitis with massive fibrosis and the presence of pseudocysts and/or stones was found in 50 (80.6%) patients; changes in the orthotopic pancreas were moderate in 10 (16%) patients and mild in two cases (3.2%).

### 3.1. Imaging and Endoscopy

**Figure 11:**
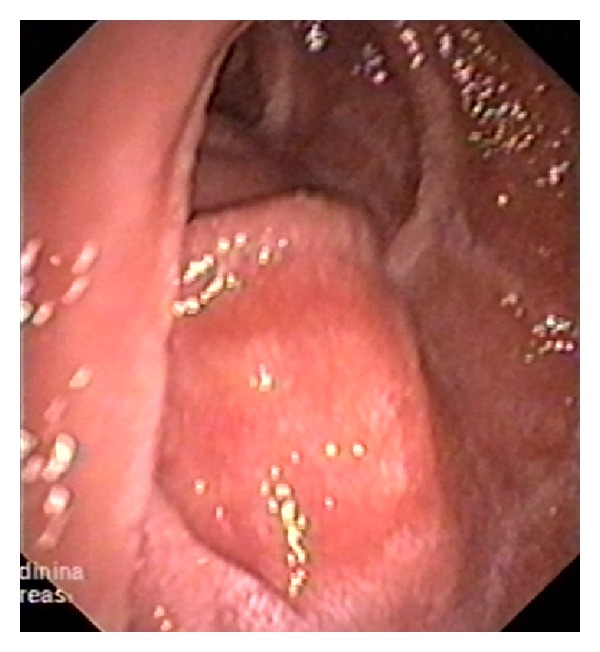
Duodenoscopy. Patient 47 y.o. Duodenal dystrophy. Cyst embedded in the medial wall of duodenum causing intrinsic contour bulge.

**Figure 12:**
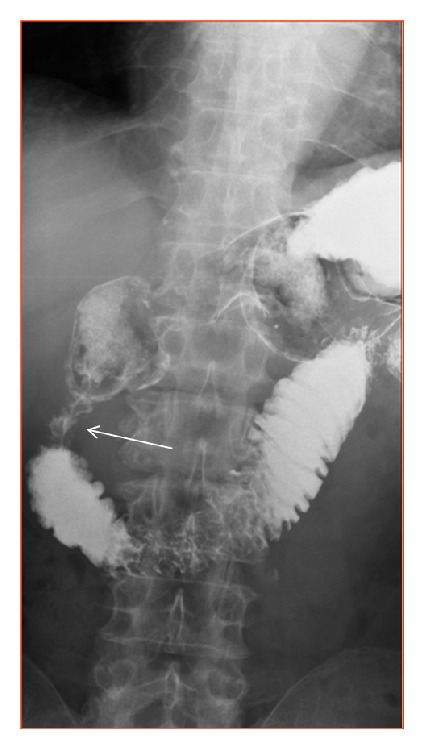
Stomach and duodenum X-ray with barium contrast. Patient 51 y.o. Duodenal dystrophy. Stenosed vertical branch of the duodenum (arrow).

The use of various procedures is given in Table 3. Abdominal ultrasound and computed tomography with intravenous contrast as well as esophagogastroduodenoscopy were performed in all patients. Only in two cases, duodenal dystrophy was suspected based on transabdominal ultrasound findings. In all cases, intrinsic contour bulge of the medial duodenal wall into the lumen was found (Figure 11), and it was associated with significant duodenal stenosis in 21 (33.6%) patients. In three patients (4.8%), the duodenal portion downstream stenosis could not be reached by the endoscope. In 20 (32%) patients, the following conditions were also found: erosive esophagitis in 11 (17.6%) patients, erosive and ulcerative duodenitis in 7 (11.2%), and erosive gastritis in 12 (19.2%) patients. X-ray examination of the stomach showed evidence of severe stenosis with stomach dilation in 8 (12.8%) patients (Figure 12).

**Figure 13:**
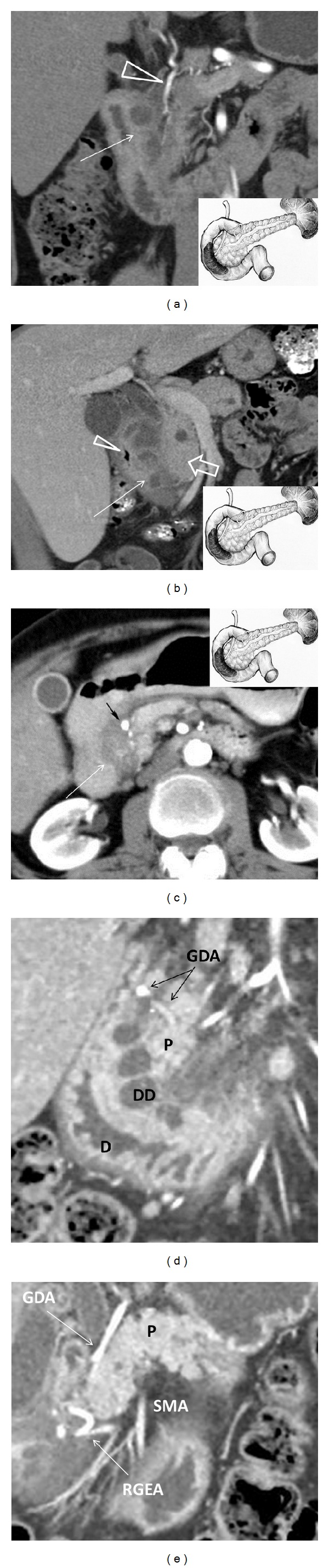
Duodenal dystrophy with moderate chronic orthotopic pancreatitis. Multidetector computed tomography. Frontal view. Patient, 32 y.o. (a) Arterial phase. Deformation and thickening of the medial wall of the duodenum with septated cyst (arrow). The gastroduodenal artery is shifted forward and to the left, lying in the groove between the pancreatic head and affected duodenal wall (arrowhead). The scheme of the lesion and the unaffected main pancreas is in the lower right corner; (b) patient 44 y.o. Venous phase. Deformed and thickened medial duodenal wall with multiple cysts (arrow), separated from moderately changed pancreatic head (thick arrow), is narrowing the duodenal lumen (arrowhead). The scheme of the lesion and the unaffected main pancreas is in the lower right corner; (c) patient 49 y.o. Arterial phase. Deformation and thickening of the medial wall of the duodenum with contrasted pancreatic tissue inside (arrow). The gastroduodenal artery is shifted forward and to the left, lying in the groove between the pancreatic head and affected duodenal wall (black arrow). The scheme of the lesion and the unaffected main pancreas is in the upper right corner. (d) Isolated form of the duodenal dystrophy. Multidetector computed tomography. (d) Male, 57 y.o. Arterial phase. Sagittal view. Deformation and thickening of the medial wall of the duodenum (D) with septated cysts (DD). The gastroduodenal artery (GDA) is shifted forward and to the left, lying in the groove between the unaffected pancreatic head (P) and duodenal wall. (e) Isolated form of the duodenal dystrophy with unchanged orthotopic pancreas. (a) Male 57 y.o. Arterial phase. Sagittal view. Septated cysts in the submucosa and muscularis of the diffusely thickened duodenal wall surround the major papilla.

**Figure 14:**
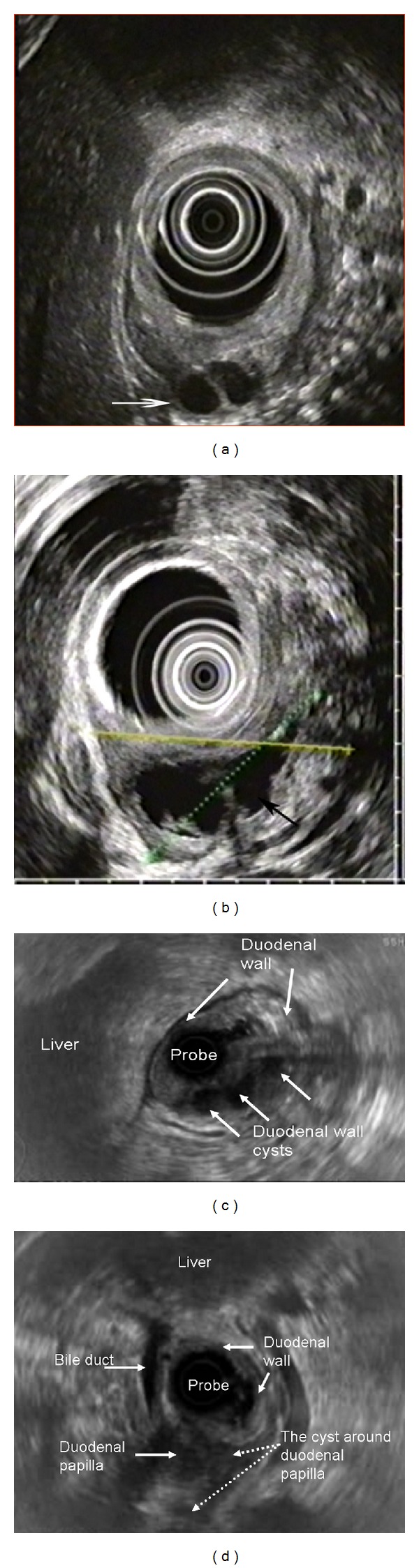
Endosonography. Duodenal dystrophy with moderate chronic orthotopic pancreatitis. (a) Patient 32 y.o. Ovoid septated cystic structure in the submucosa and muscularis of the diffusely thickened duodenal wall (arrow); (b) patient 43 y.o. Large septated multiloculated cyst in the submucosa and muscularis of the diffusely thickened duodenal wall (arrow). (c) Male 57 y.o. Isolated form of the duodenal dystrophy with unchanged orthotopic pancreas. Septated cystic structure (three arrows) in the submucosa and muscularis of the diffusely thickened duodenal wall (two arrows); (d) male, 57 y.o. Isolated form of the duodenal dystrophy with unchanged orthotopic pancreas. Septated cysts in the submucosa and muscularis of the diffusely thickened duodenal wall surround the major papilla.

MRI and MRCP were performed in 26 (41.6%) patients. The main CT and MRI findings in DD patients included thickening, infiltration, and cystic structures in the duodenal wall (Figures 13(a)–13(e)). Endoscopic ultrasound (EUS) was performed in 40 (64%) patients, and the main signs of DD were duodenal wall thickening and presence of hypoechoic cavities (100%) in the muscular and/or submucosal layer of the duodenal wall (Figures 14(a)–14(d)) [32]. The sensitivity of CT, MRI, and EUS was 95%, 84%, and 94%, and specificity was 94%, 86%, and 98%, respectively.

Signs of chronic pancreatitis in the orthotopic gland, such as calcificates, tissue heterogeneity, cysts, enlarged pancreatic head, MPD, and common bile duct dilation, were found in 50 (80.6%) patients. Cystic lesions in the head of the pancreas were found in 16 (26%) patients and the diagnosis of them was “duodenal dystrophy associated with chronic pancreatitis of the orthotopic gland.”

### 3.2. Patients and Procedures

Before surgery, all patients were observed by the gastroenterologist and received treatment for chronic pancreatitis. Before PD, four patients were subjected to ultrasound guided cholecystostomy for obstructive jaundice, and one patient underwent EUS-assisted transduodenal stenting of the duodenal wall cyst. Surgery was proposed to all patients. Ten patients refused for various reasons and continued conservative therapy under supervision of a gastroenterologist. Only 3 of 10 patients demonstrated improvement on conservative therapy and dietary restrictions, but pain episodes were not completely controlled in all patients. Indications for 52 patients who had undergone surgery are presented in Table 2. The type of proposed interventions changed as the surgeons were becoming aware that DD constituted a separate pathological entity which must be eliminated to obviate symptoms. This explains the use of draining procedures and duodenum-preserving pancreatic head resections (DPPHR) at the beginning of this study. Internal drainage, that is, pancreaticojejunal and cysto pancreaticojejunal anastomoses, was performed in 8 cases. Patients presenting severe chronic pancreatitis of the orthotopic gland were subjected to PD (24 patients) or Nakao procedures (5 patients). DPPHR using Berne or Frey modifications was performed in 5 patients. Pancreas-preserving duodenal resection (PPDR) was performed in 10 patients without or with moderate changes in the orthotopic gland: distal gastrectomy for DD in the first portion of duodenum with an extension to the pylorus and antrum (1 patient); resection of the second part of the duodenum for cyst localized in the anterolateral wall of the second portion of the duodenum (2 patients); resection of the second duodenal portion with duodeno-duodeno anastomosis (2 patients); subtotal duodenectomy (3 patients), and resection of the vertical duodenal branch with jejunal interposition (2 patients). In two patients, subtotal duodenectomy was completed by end-to-end duodenojejunal anastomosis, and in one patient, with duodenal cyst spreading to the antrum, subtotal duodenectomy was added by distal gastrectomy and completed as Roux-en-Y. One PPDR was performed for a patient who had no pain relief after previous gastroentero- and pancreatoenterostomy (Table 4).

There was no postoperative mortality. In one patient, intraoperative (PD) electrical injury of the ureter encased in the massive retroperitoneal fibrosis occurred. It required ureter stenting and ureteropelvic segment reconstruction which was successfully performed 3 months later. In one case, Nakao operation was converted into PD because of duodenal necrosis. Three episodes of bile leakage after duodenal resection were reported. It was prolonged in one case because of the leakage of the proximal duodenojejunal anastomosis. The complication was successfully treated by distal gastrectomy. Three cases of diabetes mellitus and three cases of steatorrhea were reported 12 months after PDs.

All patients were followed up. One 67-year-old woman died from the metastatic pancreatic cancer developed in the duodenal dystrophy five years after draining procedure. One patient died for an unknown reason 3.5 years after PD. About 80% of patients after PD and 90% patients after the pancreas-preserving duodenectomy reported resolution of symptoms. The duodenum-preserving pancreatic head resections and draining procedures appeared less effective. Six (20.6%) patients after PD, one (10%) after the pancreas-preserving duodenal resection, two (40%) after duodenum-preserving resection of the pancreatic head, and two (25%) patients after internal drainage reported diminished pain intensity, change in pain localization, and less often pain episodes, whereas 6 patients after draining procedures and one after DPPHR noticed no dynamics in symptoms (Tables 4 and 5).

**Figure 15:**
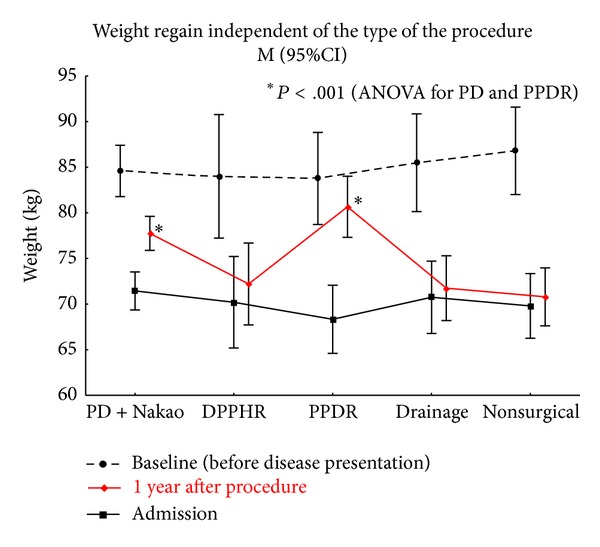
Body weight regain by the procedure performed for duodenal dystrophy. *Statistically significant difference.

Pain elimination and body weight gain is considered the most reliable objective criterion of the effectiveness of treatment option for chronic pancreatitis. Whipple procedures (PD + Nakao) and pancreas-preserving duodenal resections were significantly more efficient when compared to other treatment modalities for pain elimination and weight gain (Tables 4 and 5, Figure 15). No statistically significant difference was found between these two procedures and DPPHR with regard to pain management, probably due to a small series of pancreatic head resection: we do not use this method anymore for DD treatment, because no pain relief was achieved in 3 of 5 operated patients.

**Figure 16:**
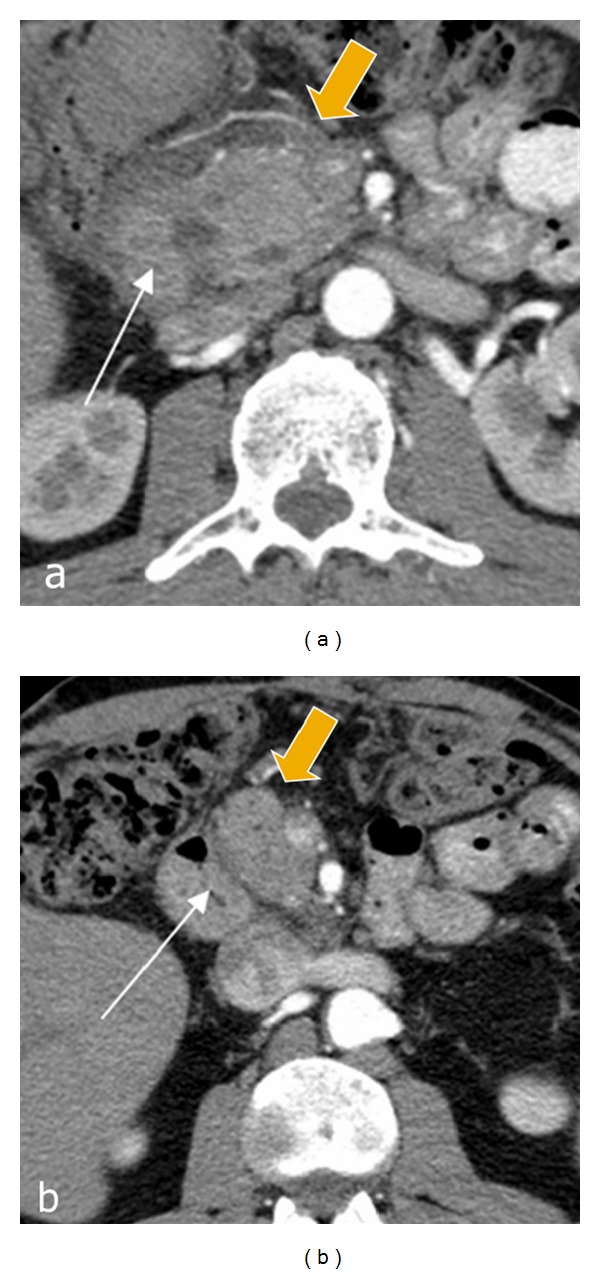
Duodenal dystrophy with moderate chronic orthotopic pancreatitis. Patient, 32 y.o. Arterial phase of multidetector computed tomography before (a) and 6 months after (b) the pancreas-preserving resection of the second portion of the duodenum with the jejunal interposition. (a) There is a septated cystic structure (thin arrow) in the medial duodenal wall with prominent inflammation and fibrosis around the duodenum and pancreatic head (thick arrow). (b) Neoduodenum (thin arrow) and pancreatic head (thick arrow) without signs of inflammation or fibrosis.

**Figure 17:**
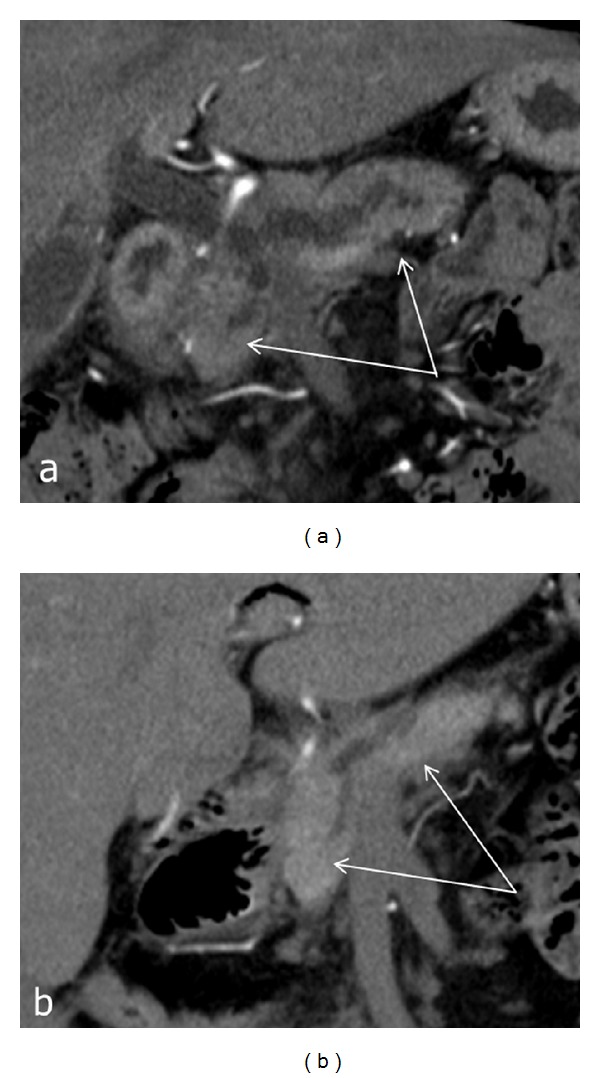
Duodenal dystrophy with moderate chronic orthotopic pancreatitis. Patient, 35 y.o. Arterial phase of multidetector computed tomography before (a) and 6 months after (b) the pancreas-preserving resection of the second portion of the duodenum with the jejunal interposition. (a) Dilation of the pancreatic and bile ducts on the background of chronic inflammation and compression of pancreatic parenchyma (arrow). (b) Narrowing of the dilated pancreatic duct and reduction of inflammation in the pancreatic head and body (arrow).

**Figure 18:**
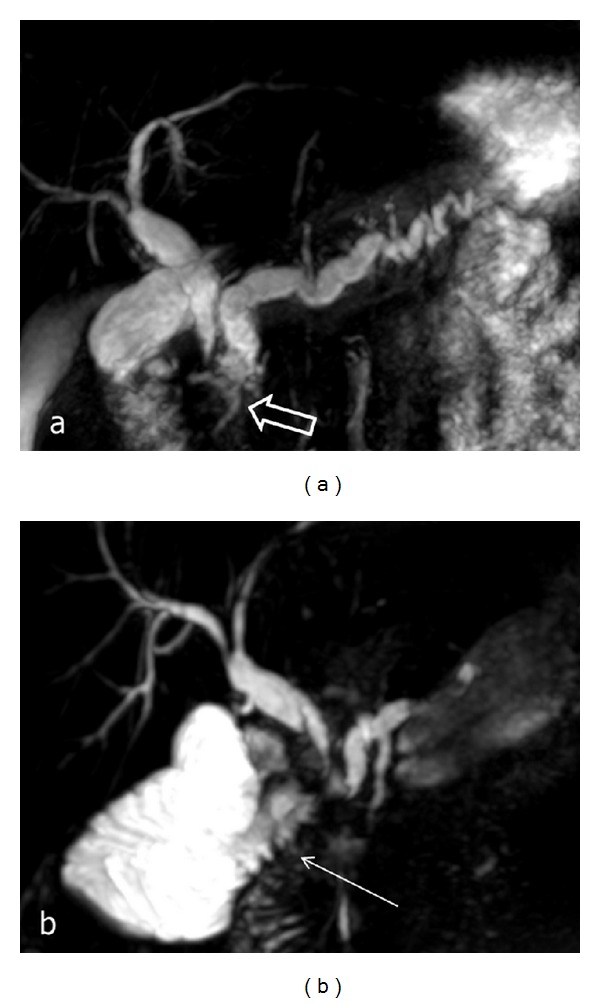
Duodenal dystrophy with moderate chronic orthotopic pancreatitis. Patient, 35 y.o. Magnetic resonance cholangiopancreatography; (a) a septated cyst is located in the medial wall of the second part of the duodenum (thick arrows) causing stenosis of the terminal parts of the common and the main pancreatic ducts with subsequent biliary and pancreatic hypertension; (b) 6 months after the pancreas-preserving resection of the second portion of the duodenum with the jejunal interposition (arrow). Narrowing of the pancreatic and common bile ducts after surgery.

CT examination performed at different times after PD, Nakao operation, and duodenum-preserving pancreatic head resection did not reveal any significant changes in the pancreatic remnant. In all cases of pancreas-preserving duodenal resections, CT scans revealed either significant reduction or disappearance of inflammation in the orthotopic pancreas (Figures 16, 17, and 18). In all other cases, prominent signs of chronic pancreatitis persisted in the orthotopic and ectopic glands, sometimes with de novo formation or increased number of stones in the parenchyma of the main gland.

## 4. Discussion 

To discuss the problem it is necessary to clarify the terms we use. Recently, Adsay and Zamboni [5], after studying of 21 pancreaticoduodenal specimens obtained from patients with chronic pancreatitis, in which chronic inflammation “predominantly involved the duodenal wall, extending to the adjacent pancreas and common bile duct,” and “…predominant pathologic process contained acinar lobules as well as pancreatic-type ducts…,” combined a number of pathological conditions previously described as cystic dystrophy in heterotopic pancreas or duodenal dystrophy [1, 4, 10, 41, 42], groove pancreatitis [43–46], pancreatic hamartoma of duodenum [47–50], paraduodenal wall cyst [8, 51–53], and myoadenomatosis [54, 55] under a new umbrella term “paraduodenal pancreatitis.” According to these authors, all the above names describe a form of chronic pancreatitis involving the duodenal wall in close proximity to the minor duodenal papilla, that is, in a so called “groove” area—the parenchymal pancreatic tissue just in between the duodenal wall and the main biliary intrapancreatic tract [4, 41–44]. Authors suggested the following pathogenetic mechanisms: for some reasons, mostly due to regular alcohol consumption and smoking, inflammation occurs in the “groove” pancreatic tissue that is clinically manifested in pain episodes resembling those in acute or chronic pancreatitis. During acute inflammatory phase, intramural areas of cystic degeneration are formed in the duodenal wall which can mimic one or more pseudocysts in the pancreatic head area adjacent to the duodenum. Eventually, owing to a typical localization of the “groove” pancreatic tissue in close proximity of the medial duodenal wall, the periampular area is becoming involved in the inflammation leading to the obstruction of the main pancreatic duct and development of chronic pancreatitis in the main (orthotopic) pancreas [5, 42, 43].

Initially, we had about the same view on such cases, and, as is clear from our series, tried to treat the disease performing surgeries that are commonly used for chronic pancreatitis, that is, draining procedure, duodenum-preserving pancreatic head resection, and Whipple procedure, more so that our patients usually had advanced diseases with fibrotic changes in the whole pancreas. With time, however, some cases accumulated, in which chronic pancreatitis involved the duodenal wall only with no or minor changes in the main (orthotopic) pancreas. In view of this, we suggested that this condition is an early stage of “paraduodenal pancreatitis,” but at the same time it meant that lesion is of “duodenal,” but not “paraduodenal,” origin, as it was concluded in the first description of the entity and by some other authors, who used the term “duodenal dystrophy” [1, 4, 7, 30]. Pathohistological study confirmed that the site of the disease is a duodenal wall, and separation of the duodenal pancreatic tissue from the main pancreas suggested its ectopic origin. It happened that the historical term “duodenal dystrophy,” not reflecting the nature of the disease, accurately demonstrates its localization. Based on histopathology findings and efficacy of pancreas-sparing duodenal resections, we consider duodenal dystrophy as a chronic inflammation of the ectopic pancreatic tissue in the duodenal wall. It can be either isolated [1, 4, 7, 30] or associated with severe inflammation and fibrosis in the main pancreas. It was precisely that circumstance that made us choose the term “duodenal dystrophy” and not “paraduodenal pancreatitis.”

As for the term “paraduodenal pancreatitis,” we think that it may be used only in cases of advanced inflammation and fibrosis of the main pancreas in presence of duodenal wall cysts, so far as, first, the term corresponds to the described condition and, second, dictates the only efficient surgical decision, that is, the Whipple procedure. Anyway, to escape confusion we prefer the definition “chronic pancreatitis associated with duodenal dystrophy” for such a combination.

Literature review confirmed our data suggesting that isolated involvement of the pancreatic tissue in the duodenum occurs in 25–30% of cases [4, 7, 30]. In the other 70–75% of cases inflammation and scarring involve the main pancreas with the progression of the disease initially located in the duodenum, and we followed up two such patients who preferred conservative treatment to surgery.

If the duodenum is only involved, resection of the main pancreas is an excessive and unnecessary option because of significant portion of the normally functioning pancreas is removed, draining procedures and head resections are inapplicable because of narrow pancreatic duct and almost unchanged parenchyma. Based on these considerations, we proposed to perform (and now consider an indication for) pancreas-preserving procedures instead of PDs in cases of isolated forms of duodenal dystrophy, when preoperative examination definitely showed preserved structure of the orthotopic pancreas without cysts, stones, and fibrosis. It makes it important to differentiate between duodenal dystrophy associated with chronic pancreatitis of the main pancreas and isolated duodenal dystrophy, so as Whipple procedure is the optimal treatment option for the former, while pancreas-preserving duodenal resection is the best surgical option for the latter.

The duodenal dystrophy among patients with chronic pancreatitis was revealed in different studies in 2.7%–25% of cases [7, 9, 18, 19, 44, 56–59], although the histological verification of DD diagnosis was seldom presented. The prevalence of DD on the background of orthotopic pancreatitis may be underestimated due to dominating symptoms of classical chronic pancreatitis and insufficient awareness of radiologists and surgeons of DD semiotics or the very fact of its existence.

As of now, this study of 62 patients comprises one of the largest series of patients with cystic form of DD [2, 4, 7, 33]. Our patients presented with abdominal pain (100% patients) and weight loss (over 75%) was elsewhere reported. Their demographic characteristics (predominantly males, mean age 45 years) were also similar [2, 4, 7, 33–36, 57]. At the time of diagnosis, nearly all patients received treatment for chronic pancreatitis. In most cases, the disease affected men who regularly consumed alcohol (95% patients in our series versus 80% reported in the literature) [9, 57–59].

Computed tomography (CT), endoscopic ultrasound (EUS), and magnetic resonance imaging (MRI) appeared the most accurate methods for DD diagnostics in our study and elsewhere [9–13]. All the methods demonstrated equally high sensitivity and specificity. Transabdominal ultrasound examination is of limited value, as it usually does not allow differentiation DD from the pancreatic head cyst. Despite early reports on insufficient sensitivity of CT for DD diagnosis [12], now we have every reason to speak about typical CT semiotics of DD [10–13]. Given high diagnostic accuracy of these methods, we can rely on them even when no morphological data are available to confirm the diagnosis. The same approach is adopted by French authors who had reported the majority of DD cases [7].

Native CT and MRI can detect only overall increase of the pancreatic head in size, which is often associated with duodenal stenosis and gastric dilation. Following contrast enhancement, fibrotic thickening of the duodenal wall is detected as areas of lower density on CT scans or lower intensity on T2-weighted MRI pulse sequences. During the venous and late phases, contrast enhancement of the pathological focus is reduced, so the density of the pathological tissue on CT scan and signal intensity on MRI are higher as compared to those of the normal pancreatic parenchyma [20]. Some authors consider the mural fibrotic induration viewed as a compact tissue layer between the duodenal lumen and pancreas the specific CT and MRI sign of DD [10]. In this area of thickened duodenal wall, one can see cysts localized in the space between the pancreatic head and vertical branch of the duodenum. These cysts are usually multiple (3 to 10), sized from 2 to 150 mm, and, unlike the pancreatic pseudocysts, they are multiloculated (more often two-chambered) and elongated (Figure 1). As the cysts are located within the duodenal wall, gradual increase in their size can lead to the shift of the gastroduodenal artery ventrally and to the left (centripetally), whereas the pancreatic head cyst displaces the artery dorsally and to the right. The solid type of duodenal dystrophy is visualized as a fibrotic thickening of the duodenal wall which is hardly differentiated from the pancreatic head. Thus, the diagnosis remains ambiguous without histological examination. For this reason, we avoided diagnosing fibrotic type of DD. In order to verify the diagnosis of DD and to personalize surgical strategy, we performed endoUS after CT scanning and/or MRI whenever possible followed by comprehensive data analysis.

Abstinence from alcohol is critical for successful treatment of DD. This is the only issue on the DD management that has been unanimously agreed on. Otherwise, expert opinions are controversial. Some authors reported successful use of somatostatin analogues [22, 39, 42, 50], whereas others [18, 19] observed no therapeutic effect of these agents. This can be due to short follow-up periods after octreotide therapy [36, 55] or lack of reports on long-term outcomes, for example, as in a large series of 105 patients, including 9 patients treated with octreotide, observed by Rebours et al. [7]. In Vankemmel et al. study [30], 1 of 7 patients treated with octreotide demonstrated a sustained, 87-month remission, 6 patients experienced recurrent symptoms within 0 to 25 months after drug withdrawal, and 5 of them were subjected to surgery. After failure of conservative therapy, minimally invasive surgical procedures were performed similarly to those used for the treatment of pancreatic pseudocysts, that is, endoscopic cystogastrostomy [55], fenestration [56], and endoscopic and percutaneous cyst aspiration with the success rate of less than 50% and follow-up periods of up to 38 months. In the study by Vankemmel et al. [30], two patients had undergone endoscopic cystoduodenostomy followed by PD after 18 and 20 months due to recurrent symptoms. Vankemmel et al. [30] suggest that endoscopic procedures are of no therapeutic value, if lesions cannot be removed, and are justified only when one or two very large cysts are present in the duodenal wall or the diagnosis of DD has to be verified histologically. The PD was the operation of choice for DD patients. However, with improved quality of preoperative diagnosis, new treatment options are evolving. Rebours et al. [7] reported that only 27% of 105 DD patients had undergone surgery, including PD in 2/3 of cases and bypass operation in the rest of cases. Vankemmel et al. [30] reported a series of 23 patients of whom 14 had undergone surgery, including PD in 12 cases (after failure of conservative and endoscopic treatment), and found no recurrences within up to 47 months postoperatively. In one case, symptoms had recurred after cystoenterostomy, and in the other case symptoms had recurred within 55 months after double bypass surgery. The majority of authors agree that the use of octreotide and its analogues, endoscopic interventions, and cyst aspiration do not guarantee long-term elimination of symptoms in patients with duodenal dystrophy, and PD remains the most effective method of DD management [25–35], although entailing high risk of postoperative endo- and exocrine pancreatic insufficiency. More conservative surgical interventions, such as pancreatic head resection and suprapapillary segmental resection of the duodenum, have been reported [27, 60] as case reports.

Our observations demonstrate that the cystic form of duodenal dystrophy can be reliably diagnosed preoperatively using up-to-date diagnostic methods and they confirm that the affected part of the duodenum must be surgically removed. Primary localization of the lesion is critical for the choice of treatment strategy and type of surgery. Dealing with this disease, we first assumed that the primary locus of pathology lies in the area of the minor duodenal papilla and is associated with the main pancreas [61]. This explains the use of draining procedures and duodenum-preserving resections of the head of the pancreas during early period of our study. These interventions, however, were not effective, pain and other symptoms relapsed very often. Surgical treatment was provided to 84% of patients after failed conservative therapy. Seventy five percent of surgical interventions involved resection and in 40 cases was associated with severe orthotopic pancreatitis, PD or its Nakao modification was the operation of choice.

Pathomorphological findings in the resected tissues, particularly in specimens with moderate inflammation, strongly convinced us that the primary lesion is the pancreatic ectopy into the duodenal wall but not an inflammation in a part of the orthotopic gland. Time of symptomatic manifestation probably depends on the distance between inflamed ectopic tissue and major papilla: when the distance is great, symptoms of main pancreatic duct obstruction appear at later terms. This can underlie the development of orthotopic pancreatitis in two patients at the age of 73 years, one of whom was a woman with no history of alcohol consumption. DD patients demonstrated resolution or significant improvement of symptoms and significantly higher body weight gain only after the resectional surgery that involved removal of the ectopic pancreatic tissue, although 17% of patients developed steatorrhea and/or diabetes mellitus 1 year after PD. The pancreas-preserving duodenal resections for DD were implemented in 2009 [62] and that was a conceptually new method of treatment targeted only at the duodenum as the primary site of the problem. Absence of diabetes and good dynamics of weight gain in patients following pancreas-preserving duodenal resections can be explained by minimal parenchyma loss caused by the pancreas preservation and absence or reversibility of orthotopic pancreatitis. Technical complexity of such procedures is comparable to that of PD, but their obvious advantage is the minimal risk for endocrine and exocrine insufficiency. Absence of symptoms in 90% of patients within 12 to 56 months postoperatively, rapid weight gain, regression of inflammation, and narrowing of the main pancreatic and common bile ducts on CT and MRCP (Figures 13–15) attest to the effectiveness of surgical treatment of DD by the duodenectomy.

The efficacy of pancreas-preserving duodenal resections for duodenal dystrophy strongly indicates that the disease affects the duodenum but not the orthotopic gland. This, in turn, translates in the following conclusions: (1) DD is a “duodenal” but not a “paraduodenal” lesion; (2) DD patients can be offered a surgical operation, which is more effective whilst less destructive than PD; and (3) gastroenterologists, radiologists, and surgeons are to be encouraged to diagnose this disease at earlier stages.

*P.S*. In two more patients successful pancreas-preserving duodenal resection was performed few months ago but results will be assessed after one-year follow-up.

The composition of patients groups is constantly changing. A few months ago, two patients from the group of conservative treatment have undergone PD because of persistent intractable pain and ineffective analgesia and severe changes of the orthotopic pancreas, and one patient was moved to the group of draining procedures because of jaundice; hence, she is scheduled for PD as well.

## Figures and Tables

**Table 1:** Characteristics of patients with duodenal dystrophy.

	Procedure					
	PD + Nakao *n* = 29	DPPHR *n* = 5	PPDR *n* = 10	Draining procedures *n* = 8	Conservative treatment *n* = 10	All *n* = 62
Age	45 (39–55)	40 (39–45)	48 (44–51)	48 (45–52)	44 (37–51)	46 (39–52)
Alcohol consumption before disease onset (mL)	72 (70–72)	72 (72-72)	68.5 (54–74)	66 (60–72.75)	72 (63–75)	72 (60–72)
Body mass before disease onset	82 (78–88)	84 (83–85)	84 (81–86)	86 (82–89)	89 (81–92)	84 (80–89)
Body mass at presentation	72 (68–78)	69 (68–73)	67 (65–71)	70 (69–74)	71 (68–72)	70 (67–75)
Body mass after surgery	78 (75–80)	73 (71–74)	80 (78–83)	72 (70–73)	71 (69–73)	75 (71–79)

The values are presented as medians with interquartile ranges (in brackets). PD: pancreaticoduodenectomy, Nakao: Nakao procedure, DPPHR: duodenum-preserving pancreatic head resection, PPDR: pancreas-preserving duodenal resections.

**Table 2:** Prevalence of symptoms at presentation.

Abdominal pain	62 (100%)
Jaundice	10 (16%)
Vomiting/duodenal obstruction	20 (32%)
Weight loss	56 (90%)
Tumor suspicion	2 (3.2%)

**Table 3:** Methods used for duodenal dystrophy diagnostics.

Transabdominal ultrasound	100%
MDCT	100%
MRI + MRCP	42%
Endoscopic ultrasound	66%

MDCT: multidetector computed tomography, MRI: magnet-resonance imaging, MRCP: magnet-resonance cholangiopancreatography.

**Table 4:** Results of surgery for duodenal dystrophy.

Procedure	Number	Morbidity	Full symptoms elimination	Steatorrhea	New diabetes mellitus
PD + Nakao	29	5 (17%)	23 (79%)	4 (14%)	3 (10%)
DPPHR	5	3	2	—	—
Internal drainage	8	2	2	1	1

Pancreas-preserving duodenal resections (PPDR)					
With direct duodeno-duodenoanastomosis	4	2	3	—	—
Subtotal duodenectomy	3	—	3	—	—
With intestinal interposition	2	1	2	—	—
Distal gastrectomy	1	—	1	—	—
All PPDRs	10	3	9	—	—

PD: pancreaticoduodenectomy, Nakao: Nakao procedure, DPPHR: duodenum-preserving pancreatic head resection.

**Table 5:** Efficacy of the PPDR and other treatment options for pain elimination in case of duodenal dystrophy.

Group	No pain	Have pain	Total *N*	*P* value
PPDR	9	1	10	

vs				
DPPHR	2	3	5	0.076
PD + Nakao	23	6	29	0.652
Draining procedure	2	6	8	**0.012**
Consevative treatment	2	8	10	**0.005**

PD: pancreaticoduodenectomy, Nakao: Nakao procedure, DPPHR: duodenum-preserving pancreatic head resection, PPDR: Pancreas-preserving duodenal resections. Fisher's exact test two-sided (or two-tailed), *P* values < 0.05 mean a significant difference.
